# Minimally invasive, imaging guided virtual autopsy compared to conventional autopsy in foetal, newborn and infant cases: study protocol for the paediatric virtual autopsy trial

**DOI:** 10.1186/1471-2431-14-15

**Published:** 2014-01-20

**Authors:** Christoph M Rüegger, Christine Bartsch, Rosa Maria Martinez, Steffen Ross, Stephan A Bolliger, Brigitte Koller, Leonhard Held, Elisabeth Bruder, Peter Karl Bode, Rosmarie Caduff, Bernhard Frey, Leonhard Schäffer, Hans Ulrich Bucher

**Affiliations:** 1 Department of Neonatology, University Hospital Zurich, Zurich, Switzerland; 2 University Children’s Hospital, Zurich, Switzerland; 3 Institute of Forensic Medicine, University of Zurich, Zurich, Switzerland; 4 Division of Biostatistics, University of Zurich, Zurich, Switzerland; 5 Institute for Pathology, University Hospital Basel, Basel, Switzerland; 6 Department of Pathology, University Hospital Zurich, Zurich, Switzerland; 7 Division of Obstetrics, University Hospital Zurich, Zurich, Switzerland

**Keywords:** Autopsy, Post-mortem imaging, Minimally invasive virtual autopsy, Guided biopsy, Virtopsy®, Foetus, Stillbirth, Newborn, Infant

## Abstract

**Background:**

In light of declining autopsy rates around the world, post-mortem MR imaging is a promising alternative to conventional autopsy in the investigation of infant death. A major drawback of this non-invasive autopsy approach is the fact that histopathological and microbiological examination of the tissue is not possible. The objective of this prospective study is to compare the performance of minimally invasive, virtual autopsy, including CT-guided biopsy, with conventional autopsy procedures in a paediatric population.

**Methods/Design:**

Foetuses, newborns and infants that are referred for autopsy at three different institutions associated with the University of Zurich will be eligible for recruitment. All bodies will be examined with a commercial CT and a 3 Tesla MRI scanner, masked to the results of conventional autopsy. After cross-sectional imaging, CT-guided tissue sampling will be performed by a multifunctional robotic system (Virtobot) allowing for automated post-mortem biopsies. Virtual autopsy results will be classified with regards to the likely final diagnosis and major pathological findings and compared to the results of conventional autopsy, which remains the diagnostic gold standard.

**Discussion:**

There is an urgent need for the development of alternative post-mortem examination methods, not only as a counselling tool for families and as a quality control measure for clinical diagnosis and treatment but also as an instrument to advance medical knowledge and clinical practice. This interdisciplinary study will determine whether virtual autopsy will narrow the gap in information between non-invasive and traditional autopsy procedures.

**Trial Registration:**

ClinicalTrials.gov: NCT01888380

## Background

Despite the long-standing clinical success of post-mortem examinations [1], there has been a sustained decline in autopsy rates around the world [2]. This development has been observed not only in adults, but even more frequently in neonates, infants and children [3-5]. This is in spite of the fact that perinatal and paediatric autopsies present particular value in several regards.

First, perinatal post-mortem examinations allow for the identification of genetic and obstetric factors that are of relevance to the management of future pregnancies, allowing for appropriate counselling of families. In approximately 30% of cases following termination of pregnancies, foetal autopsy changed the recurrence risk associated with those parents [6,7]. Second, foetal and paediatric autopsies are important in confirming or refuting pre-morbid diagnoses, and present information pertinent to further specific diagnoses. Stambouly et al. demonstrated that paediatric autopsies reveal additional findings in up to 50% of cases, approximately 10% of which identified diagnoses that may have prevented death had they been known [8]. Finally, in addition to the benefits to individuals and families, autopsy has the potential to advance medical knowledge and improve clinical practice [9].

The reasons for the decrease in autopsy rates are multifactorial and complex. Importantly, a large majority of parents deny consent to autopsy because of concerns about disfigurement of their deceased child during the procedure. The desire for their infant to “be left in peace” and scepticism concerning other potential benefits or detriments are additional causes for this denial [10,11]. Additionally, the controversial perception that improved technology renders the autopsy redundant may have altered the interest and attitude of the public and clinicians towards traditional post-mortem examinations.

Questions have therefore arisen regarding the merit of traditional autopsy as a means of quality control for clinical diagnosis and treatment. For this reason, many clinicians have called for a minimally invasive autopsy technique that may be better accepted by parents and healthcare professionals and could therefore provide an alternative for those parents for whom traditional autopsy is not an option. Previous studies have indicated a better acceptance of such an autopsy approach among both healthcare professionals [12] and parents [13].

Less invasive methods, such as post-mortem ultrasound- and laparoscopic examinations [14], have been reported in the last two decades. However, these methods did not gain widespread acceptance due to a lack of evidence from studies comparing such an approach with conventional autopsy in a rigorous and blinded manner. Post-mortem magnetic resonance imaging (post-mortem MRI) and computed tomography (CT), which are generically referred to as post-mortem cross-sectional imaging [15], are other non-invasive autopsy techniques first reported in 1996 [16]. The procedures have the advantage of determining the position of the different organs in situ while maintaining the integrity of the human body. Numerous studies have now investigated the diagnostic accuracy of post-mortem MRI compared to conventional autopsy. Most of them showed good results in the detection of central nervous system abnormalities, but relatively poor results for the detection of cardiac anomalies [17-21]. The most recent and so far the largest, prospective validation study in foetuses and children demonstrated an overall concordance of 89.3% regarding the cause of death or major pathological lesions detected by minimally invasive autopsy (post-mortem MRI and blood sampling) compared with conventional autopsy [22]. In children and adolescents, the concordance was generally lower than in foetuses (53.6% vs. 94.6%), mainly because of undetected sepsis affecting the lungs, the heart, and the intestine, or because of disseminated sepsis.

In such scenarios, analysis of microstructure at the electron microscopic level as well as specific staining, would be informative [23]. However, this is not possible with an imaging-based, non-invasive autopsy approach. To overcome this shortcoming, percutaneous organ biopsies with or without image guidance have been proposed [24,25]. Biopsies leave only minor lesions and enable processing of specimens in a similar manner as those obtained by conventional autopsy. Major limitations include the relatively small amount of tissue that can be collected and the risk that the best representative parts are missed. Breeze et al. investigated the feasibility of percutaneous foetal organ biopsies using ultrasound guidance ± surface landmarks in the context of a minimally invasive autopsy. They reported that this technique cannot yet be considered to provide useful clinical information, because less than 50% of all biopsies were adequate for histological examination [26]. Percutaneous needle biopsies under CT guidance are therefore suggested to be a more reliable method for targeted sampling of tissue probes, narrowing the information gap between non-invasive and traditional autopsy [27]. However, a large prospective study of such a minimally invasive autopsy approach by post-mortem cross-sectional imaging and CT-guided biopsies in foetuses, newborns and infants has not been conducted. With the proposed interdisciplinary study, we aim to compare the performance of combined cross-sectional imaging and CT-guided biopsy with conventional autopsy. For this project, an approach that combines post-mortem imaging and imaging guided biopsies is defined as minimally invasive or virtual autopsy.

## Methods/Design

### Trial design

This is a prospective, comparative, interdisciplinary, clinical trial with three study sites: the Department of Neonatology of the University Hospital Zurich, the Children’s University Hospital Zurich and the Institute of Forensic Medicine. All three study sites are associated with a single academic institution – the University of Zurich, Switzerland. Consecutive foetuses, newborns and infants that are referred for autopsy at these three institutions will be eligible for recruitment into the study. All recruited cases will undergo post-mortem imaging (MRI and CT) with CT-guided biopsies in addition to conventional autopsy.

The study protocol was submitted Ethical Committee of the Canton Zurich and received a waiver from ethics approval. Clinical trial registration was made at clinicaltrials.gov under the number NCT01888380. The study will be conducted in accordance with principles enunciated in the current version of the Declaration of Helsinki, the guidelines of Good Clinical Practice issued by the International Conference on Harmonization, and the requirements of the Swiss regulatory authority. With the exception of forensic cases, parental consent will be required for each subject.

### Inclusion and exclusion criteria

Inclusion criteria for participation in the paediatric virtual autopsy trial comprise still- and live-born infants from 16 weeks of gestational age up to 12 months postnatal age regardless of the manner of death (trauma, homicide, or intoxication). Deceased infants who are organ donors will be excluded from the study.

### Objectives

Our primary objective is to compare the accuracy of post-mortem imaging combined with guided biopsy for the detection of the cause of death and/or major pathological lesions to that of conventional autopsy in foetuses, newborns and infants. Secondary objectives primarily aim to evaluate which clinical indications are best suited to each of the two approaches. Second, we aim to optimise a protocol for MRI examinations in deceased neonates, infants and children. Third, this study aims to report the number of cases in which there is a change in the ante-mortem diagnosis following post-mortem cross-sectional imaging using CT-guided biopsy.

### Recruitment

As routinely practiced, post-mortem examinations will be offered for all foetuses, newborns and infants up to 12 months of age regardless of whether the death was due to a natural or non-natural cause. In non-medical legal cases, parents will be approached by appropriately trained staff (a consultant or an experienced midwife) and asked if they consent to an autopsy for their child. If parents agree, a precedent cross sectional imaging with CT-guided biopsy will be proposed. They will receive an information leaflet and at least one parent must give written consent.

### Post-mortem imaging

All bodies will be examined with a commercial dual-source CT (Somatom Definition; Siemens Medical Solutions, Forchheim, Germany) and a 3 Tesla MRI scanner (Philips Achieva; Philips Medical Systems, Best, The Netherlands) at the Institute of Forensic Medicine, University of Zurich. MR images will be acquired as given in Table 1 and will include the complete foetal or infant body from the calvarium through the lower extremities. The first 30 subjects will be used to improve the imaging sequences and to optimise the robotic system allowing for CT-guided tissue sampling. These subjects will not be included in the main study. Initially, a specialised forensic radiologist (S.R.) with 6 years of experience in post-mortem radiology will report the results of MR and CT imaging in a large internet-based secure database (SecuTrial®). All diagnoses will be specified according to the international classification of diseases (ICD-10).

**Table 1:** Sequences for post-mortem magnetic resonance imaging

**Sequence**	**Slice thickness (mm)**	**TR (ms)**	**TI (ms)**	**TE (ms)**	**IR delay (ms)**
**Body Imaging**					
T2 coronal	2	2969		100	
T2 SPIR coronal	2	3155		100	
T1 IR coronal	2	7000	600	15	600
T2 axial	2	4000		80	
T2 SPIR axial	2	3341		60	
T1 IR axial	2	7000	600	15	600
T2 sagittal	2	2969		100	
**Brain Imaging**					
T2 axial	2	5531		50	
FLAIR axial	2	12000	2850	140	2850
T1 IR axial	2	7000	600	15	600

*TR*, relaxation time, *TI*, inversion time, *TE*, echo time, *IR*, inversion recovery, *mm*, millimetre, *ms*, millisecond, *SPIR*, spectral presaturation with inversion recovery, *FLAIR*, fluid attenuated inversion recovery.

### CT-guided tissue sampling

After cross-sectional imaging, CT-guided tissue sampling will be performed by a multifunctional robotic system at the Institute of Forensic Medicine, University of Zurich. This “Virtobot System” allows for automated post-mortem biopsies and is composed of a six-axis industrial robotic arm (Stäubli TX90L; Stäubli, Freienbach, Switzerland) that has been mounted to an external axis, aligned with the CT table [28]. For performing biopsies, the Virtobot has been combined with a surgical navigation system. Under close monitoring by a forensic pathologist specialising in paediatric forensic medicine, the same predefined organs (surface of the brain including the meninges, upper and lower lobes of the lung, right and left lobe of the thymus, right and left heart ventricle, right and left lobe of the liver, both kidneys, adrenal glands, pancreas, and spleen) will be biopsied in each case. Additional samples will be taken if, based on imaging, a focal abnormality is suspected. Specimens will be fixated in formalin and examined by an independent pathologist, who is blinded to the results of post-mortem imaging and conventional autopsy results. Ancillary tests include the minimally invasive examination of the placenta and umbilical cord and CT-guided extraction of blood, urine, and cerebrospinal fluid for microbiological diagnostics (cultures).

### Virtual autopsy – final classification

Based on cross-sectional imaging, histology and ancillary test results, the findings will be reclassified with regard to the likely final diagnosis and major pathological findings. An interdisciplinary team (forensic radiologist, forensic pathologist and pathologist) blinded to the findings of the conventional autopsy will decide whether one or several pathological findings are present for each organ system. This final classification based on virtual autopsy will be reported in the same database and specified according to the ICD-10.

### Conventional autopsy

All conventional autopsies, the gold standard against which the virtual autopsy is assessed, will be performed by experienced perinatal, paediatric and forensic pathologists according to the guidelines of the Swiss Society of Pathology [29]. A database with independent access portals will be used to maintain blinding from virtual autopsy results. For each organ system, the same categorisation (likely final diagnosis and major pathological findings) will be used and specified according to the ICD-10.

### Sample size calculation and statistical analysis

A sample size of 100 cases allows for the computation of a 95% confidence interval of width +/− 8% for the primary outcome if the true percentage of cases for which virtual autopsy correctly identifies the diagnostic category is 80%. The width of the confidence interval will decrease to +/− 6% if the true percentage is 90%, and further decrease to +/− 4% if the true percentage is 95%. This precision is considered as sufficient to determine the quality of virtual autopsy.

The diagnoses established by the two methods and by the two independent teams will be compared, with the autopsy results considered the gold standard. The primary outcome will be the percentage of cases for which virtual autopsy correctly identifies the diagnostic category, which will be given together with a 95% confidence interval. Sensitivity, specificity and predictive values will be calculated for each diagnostic category, as referenced to the gold standard, again with a 95% confidence interval. Because of the small sample sizes, the confidence interval by Wilson will be used throughout, as recommended in Altman et al. in Chapters 6 and 10 [30]. Finally, the results of the virtual autopsy will be assessed with regard to the accuracy of associated lesions, the clinical utility of the information and the determination of the cause of death.

## Discussion

Based on previous research, post-mortem MRI appears to be a promising alternative to conventional autopsy in foetuses, newborns and infants. The only disadvantage of the imaging based autopsy is the fact that tissue for histopathological and microbiological examination cannot be collected, resulting in a poor accuracy of MRI due to the fact that infection is a primary cause of death in older infants and children.

This study protocol describes the design of a new paediatric virtual autopsy trial, which employs a minimally invasive autopsy procedure that includes tissue sampling with the aid of a robotic system. To the best of our knowledge, this is the first prospective study to evaluate the accuracy of such a virtual autopsy approach compared to conventional autopsy procedures in a paediatric population.

## Abbreviations

CT: Computed tomography; ICD: International classification of diseases; MRI: Magnetic resonance imaging.

## Competing interests

The authors declare that they have no competing interests.

## Authors’ contributions

CR is responsible for the conception, design and conduct of the study. CR developed a data collection system and drafted the study protocol. CB will be responsible for the minimally invasive virtual autopsy. SR will interpret the cross-sectional imaging data and report together with CB, RM, and SAB the final classification based on virtual autopsy. BK will give administrative support. LH will be responsible for the statistical analyses. EB will process the tissue derived from virtual autopsies and will report the results of the histological examination. PKB and RC will perform conventional autopsies. BF and LS provided input into the clinical aspects of the study and will be responsible for the recruitment of the cases. HUB has overall responsibility for the study and advised on the conception, design and conduct of the study. He critically revised the article. All authors read and approved the final manuscript.

## Funding

C.R. was supported by a Swiss National Science Foundation (SNSF) Career Award for Medical Scientists (33CM30_140334).

## Pre-publication history

The pre-publication history for this paper can be accessed here:

http://www.biomedcentral.com/1471-2431/14/15/prepub
